# *In silico* characterization of competing endogenous RNA network in glioblastoma multiforme with a systems biology approach

**DOI:** 10.3389/fonc.2022.1024567

**Published:** 2022-10-13

**Authors:** Soudeh Ghafouri-Fard, Arash Safarzadeh, Bashdar Mahmud Hussen, Mehdi Akhavan-Bahabadi, Mohammad Taheri, Guive Sharifi

**Affiliations:** ^1^ Department of Medical Genetics, School of Medicine, Shahid Beheshti University of Medical Sciences, Tehran, Iran; ^2^ Department of Pharmacognosy, College of Pharmacy, Hawler Medical University, Erbil, Iraq; ^3^ Center of Research and Strategic Studies, Lebanese French University, Erbil, Iraq; ^4^ Electronic Engineering, University of Tehran, Tehran, Iran; ^5^ Men’s Health and Reproductive Health Research Center, Shahid Beheshti University of Medical Sciences, Tehran, Iran; ^6^ Institute of Human Genetics, Jena University Hospital, Jena, Germany; ^7^ Skull Base Research Center, Loghman Hakim Hospital, Shahid Beheshti University of Medical Sciences, Tehran, Iran

**Keywords:** glioblastoma multiforme (GBM), ceRNA, lncRNA, miRNA, biomarker

## Abstract

Glioblastoma multiforme (GBM) is the most frequent malignant type of primary brain cancers and is a malignancy with poor prognosis. Thus, it is necessary to find novel therapeutic modalities based on molecular events occur at different stages of tumor progression. We used expression profiles of GBM tissues that contained long non-coding RNA (lncRNA), microRNA (miRNA) and mRNA signatures to make putative ceRNA networks. Our strategy led to identification of 1080 DEmRNAs, including 777 downregulated DEmRNAs (such as GJB6 and SLC12A5) and 303 upregulated DEmRNAs (such as TOP2A and RRM2), 19 DElncRNAs, including 16 downregulated DElncRNAs (such as MIR7-3HG and MIR124-2HG) and 3 upregulated DElncRNAs (such as CRNDE and XIST) and 49 DEmiRNAs, including 10 downregulated DEmiRNAs (such as hsa-miR-10b-5p and hsa-miR-1290) and 39 upregulated DEmiRNAs (such as hsa-miR-219a-2-3p and hsa-miR-338-5p). We also identified DGCR5, MIAT, hsa-miR-129-5p, XIST, hsa-miR-128-3p, PART1, hsa-miR-10b-5p, LY86-AS1, CRNDE, and DLX6-AS1 as 10 hub genes in the ceRNA network. The current study provides novel insight into molecular events during GBM pathogenesis. The identified molecules can be used as therapeutic targets for GBM.

## Introduction

Glioblastoma multiforme (GBM) is the most frequent malignant type of primary brain cancers (1). This type of cancer is classified into primary and secondary subtypes based on the presence of mutations in isocitrate dehydrogenase (IDH) genes (2). Moreover, the mutation status of *IDH1* and *IDH2* genes is considered as an important factor in defining prognosis of GBM (2). GBM is a highly invasive tumor with high tendency to diffuse all over the brain parenchyma. In addition, high level of vascularity of GBM makes it exceedingly recidivist, leading to a short survival time even after surgical resection and chemoradiotherapy (2). From an immunological point of view, GBM is regarded as a cold tumor with an extremely immunosuppressive tumor microenvironment that acts in favor of tumor progression, recurrence and poor prognosis (2). Therefore, it is necessary to find novel therapeutic modalities based on molecular events occur at different stages of tumor progression.

Recent studies have used expression data of differentially expressed RNAs in GBM to construct competitive endogenous RNA (ceRNA) networks with the potential to be used as prognostic factors (3–6). In one of the recent studies, one lncRNA with differential expression related to survival, IL10RB-AS1, was discovered using a combination of bioinformatic techniques. This may have predictive utility and present novel therapy options for GBM, along with a number of associated signaling pathways and ceRNA systems that were discovered in GBM (7). This strategy can also been used to identify subtype-specific modules with distinctive biological functions that influence patients’ prognosis in different GBM subtypes (8). Therefore, it is very important to look into probable genetic causes of GBM. One of the most urgent difficulties in cancer therapy is the creation of alternate and acceptable biomarkers to accurately identify and treat GBM (9).

In the current study, we used expression profiles of GBM tissues that contained long non-coding RNA (lncRNA), microRNA (miRNA) and mRNA signatures to make putative ceRNA networks. Then, we find the molecular pathways which are associated with these ceRNA networks.

## Methods

### Microarray data collection

Expression profiles of GSE50161 ([HG-U133_Plus_2] Affymetrix Human Genome U133 Plus 2.0 Array), GSE36245 ([HG-U133_Plus_2] Affymetrix Human Genome U133 Plus 2.0 Array), GSE83300 (Agilent-014850 Whole Human Genome Microarray 4x44K G4112F (Probe Name version)) and GSE65626 ([miRNA-4] Affymetrix Multispecies miRNA-4 Array), which included 130, 46, 50 and 6 samples, respectively, were acquired using the Gene Expression Omnibus (GEO; http://www.ncbi.nlm.nih.gov/geo/). We selected 34 GBM and 13 normal tissue samples from GSE50161, 46 GBM samples from GSE36245, 50 GBM samples from GSE83300 and 3 GBM and 3 normal samples from GSE65626 for further analysis (**Table 1**). The expression data contained lncRNAs, miRNAs and mRNAs expression signatures.

**Table 1:** Information of datasets.

Datasets	Platform	Use	Patient	Control	Tissue
**GSE50161** **GSE36245** **GSE83300** **GSE65626**	GPL570 GPL570 GPL6480 GPL19117	DEmRNA – DElncRNA DEmRNA – DElncRNA DEmRNA – DElncRNA DEmiRNA	34 46 50 3	13 - - 3	Brain Brain Brain Brain

### Microarray data processing, integrative meta-analysis and assessment of data quality

The described datasets contain different and trendy platforms (Agilent and Affymetrix), and normalization is a critical step in the integration of heterogeneous data. All microarray data was processed and integrated using the statistical programming language R. For pre-processing, data from Affymetrix and Agilent was first normalized separately using the normalizeQuantiles function in the preprocessCore package (https://bioconductor.org/packages/release/bioc/html/preprocessCore.html). The program R was used to combine data from both platforms. In order to exclude batch effects (non-biological differences), the ComBat function from the R Package Surrogate Variable Analysis (SVA) was used (10). By using PCA and a boxplot, batch effect removal was evaluated. The result of the meta-analysis is a unit expression matrix (the combination of three datasets of this study).

### Analysis of differentially expressed lncRNAs, miRNAs and mRNAs

We used the Limma package in R language (11) to screen differentially expressed mRNAs (DEmRNAs), lncRNAs (DElncRNAs), and miRNAs (DEmiRNAs) between GBM and normal samples. GSE50161, GSE36245 and GSE83300 were used to obtain DEmRNAs and DElncRNAs. GSE65626 was utilized to acquire DEmiRNAs. DEmRNAs and DElncRNAs were evaluated with the cut-off criteria of false discovery rate (FDR; adjusted p value) < 0.05 and |log2 fold Change (FC)| > 2 while the cut-off criteria of false discovery rate (FDR; adjusted p value) < 0.05 and |log2 fold Change (FC)| > 3.5 was considered for DEmiRNAs. Then, we identified DElncRNAs using HGNC (HUGO gene nomenclature) database.

### Two-way clustering of DEGs

We determined gene expression levels of significant DEmRNAs, DElncRNAs, and DEmiRNAs. We used this data in the pheatmap package in R language (12) to perform two-way clustering based on the Euclidean distance.

### Gene ontology enrichment analysis

We used the clusterProfiler R package (13) to perform gene ontology (GO) enrichment analysis to investigate the functions of the remarkably upregulated and downregulated DEGs that we discovered. The functional category criteria were established at an adjusted p-value of 0.05 or below.

### Kyoto encyclopedia of genes and genomes pathway analysis

KEGG pathway analysis of considerably DEGs was carried out to discover the possible functions of these genes that participated in the pathways based on the KEGG database (14).

### PPI network construction and hub genes identification

The STRING database (15) was utilized to create the PPI network for DEGs. Highest level of confidence was used to establish the interactions parameter (confidence score >0.9). The Cytoscape software v3.9 (16) was used to visualize the interactions between the proteins. The top 20 DEGs related to hub genes were ultimately found using the Cytohubba plugin (17) of Cytoscape using the betweenness approach.

### Constructing the ceRNA network and hub genes identification

We built a ceRNA network through the following steps: 1) assessing the interactions between lncRNAs and miRNAs based on the GBM-related miRNAs using miRcode (http://www.mircode.org/); 2) Application of miRDB (http://www.mirdb.org/) (18), miRTarBase (https://mirtarbase.cuhk.edu.cn/) (19), TargetScan (http://www.targetscan.org/) (20) and miRWalk (http://129.206.7.150/) (21) for prediction of miRNAs-targeted mRNAs; 3) Finding the intersections of the differentially expressed lncRNAs and mRNAs, and establishment of lncRNA/mRNA/miRNA ceRNA network using Cytoscape v3.9 and 4) predicting hub genes using cytohubba plugin based on degree method.

### Validation of hub genes *via* expression values

The expression value of hub genes was assessed using the ualcan database (22). The hub genes in the TCGA-GBM RNA-seq data were examined, and those were present in the PPI and ceRNA networks as well as in the TCGA-GBM were chosen for gene expression validation.

### Expression of the hub genes in various GBM cell lines

We selected GBM and normal brain cell lines using cancer cell line encyclopedia (CCLE) (https://sites.broadinstitute.org/ccle/) and DepMap portal gene expression datasets (https://depmap.org/portal/). In order to determine how the hub genes are expressed, we finally employed the limma package of the R programming language to analyze this data.

### Survival analysis

We used survival package (https://CRAN.R-project.org/package=survival) in R to define survival curves, which were grouped by the prognostic value of hub genes with highest degree in ceRNA network. The clinical data for patients with GBM derived from TCGA (PRAD-TCGA). Univariate survival analysis was evaluated using Kaplan-Meier curves. Statistics were considered significant for P-values under 0.05.

## Results

### Microarray data processing, integrative meta-analysis and assessment of data quality

**Figure 1:**
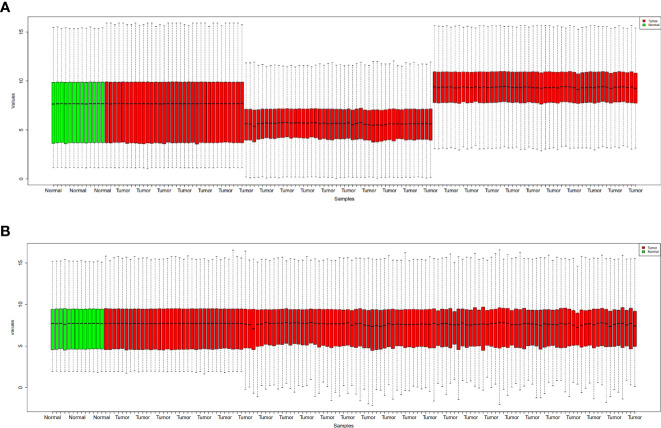
Boxplots for the raw data **(A)** and normalized data after batch effect removal **(B)**. GBM samples are indicated by red boxes, whereas healthy samples are shown by green boxes.

**Figure 2:**
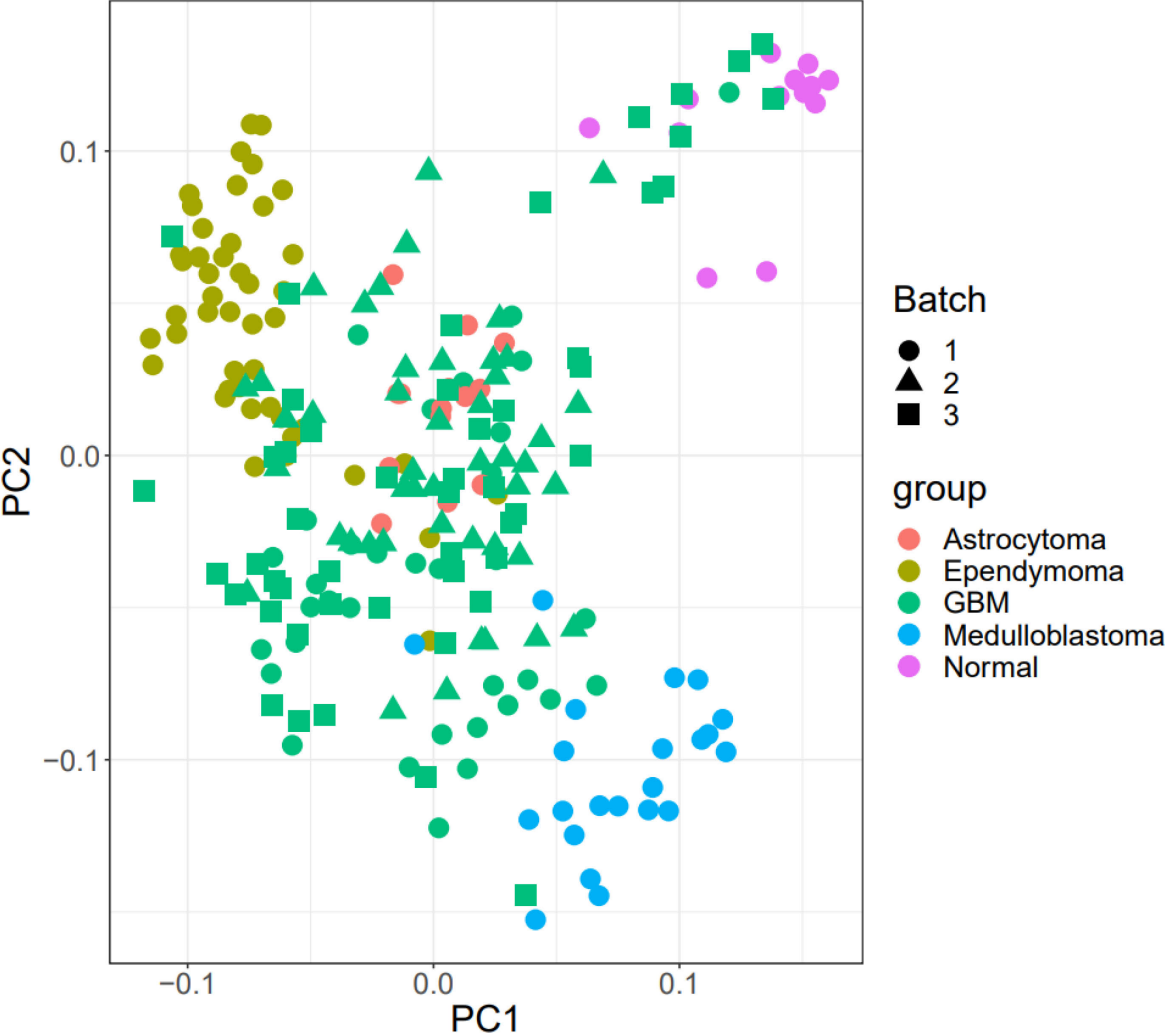
PCA plot. The Batch implies that the data includes three platforms. Also, healthy benign and tumor samples were divided into three groups.

**Figure 1** shows the boxplot of raw data and normalized data after batch effect removal. These boxplots indicate that the quality of the expression data was reliable, and the boxplot of the preprocessed data presented the good normalization. In the PCA plot (**Figure 2**), 143 samples are shown in the 2D plane spanned by their first two principal components (PC1 and PC2). According to this plot, the samples had a good variation after batch effect removal.

### DEGs analysis

Based on the microarray data analysis between GBM and normal samples using Limma package, we obtained 1080 DEmRNAs, including 777 downregulated DEmRNAs (such as GJB6 and SLC12A5) and 303 upregulated DEmRNAs (such as TOP2A and RRM2), 19 DElncRNAs, including 16 downregulated DElncRNAs (such as MIR7-3HG and MIR124-2HG) and 3 upregulated DElncRNAs (such as CRNDE and XIST) and 49 DEmiRNAs, including 10 downregulated DEmiRNAs (such as hsa-miR-10b-5p and hsa-miR-1290) and 39 upregulated DEmiRNAs (such as hsa-miR-219a-2-3p and hsa-miR-338-5p). The most significantly upregulated and downregulated DEmRNAs, DElncRNAs, and DEmiRNAs are shown in **Tables 2**–**4**, respectively.

**Table 2:** The top 10 up- and down-regulated DEmRNAs between GBM and normal samples.

Down-regulated			Up-regulated		
DEmRNA	Log FC	Adjusted *P* value	DEmRNA	Log FC	Adjusted P value
**GJB6** **SLC12A5** **PACSIN1** **SYNPR** **VSNL1** **CAMK2A** **SV2B** **NEFM** **ETNPPL** **SVOP**	-7.096688 -6.726824 -6.712373 -6.505139 -6.469234 -5.812767 -5.752584 -5.737146 -5.735033 -5.733442	3.082137e-24 3.298847e-17 1.336264e-18 2.538197e-14 2.949472e-12 8.827178e-20 1.830778e-15 5.630558e-12 5.781047e-10 6.691090e-20	**TOP2A** **RRM2** **KIAA0101** **UHRF1** **ASPM** **NUSAP1** **PBK** **WEE1** **CDK1** **CFI**	5.106081 4.991494 4.748463 4.484171 4.420167 4.218903 4.210402 4.195263 4.164108 4.054792	5.083076e-14 9.047466e-15 1.192890e-17 8.187032e-22 1.604840e-10 1.390467e-14 6.369782e-10 1.993886e-22 1.641432e-16 2.503017e-08

**Table 3:** The up- and down-regulated DElncRNAs between GBM and normal samples.

Down-regulated			Up-regulated		
DElncRNA	Log FC	Adjusted *P* value	DElncRNA	Log FC	Adjusted P value
**MIR7-3HG** **MIR124-2HG** **DGCR5** **DCTN1-AS1** **RFPL1S** **DPP10-AS1** **SLC26A4-AS1** **HAR1A** **LY86-AS1** **LINC00622** **MEG3** **MIAT** **PART1** **LINC01102** **DLX6-AS1** **TTTY15**	-3.826937 -3.483949 -3.466361 -3.326676 -3.284252 -3.174864 -2.994545 -2.760872 -2.705636 -2.67454 -2.551883 -2.33424 -2.318588 -2.121837 -2.06398 -2.01289	2.943952e-14 3.114815e-10 2.495893e-19 1.312921e-23 1.929947e-12 3.760156e-08 1.566864e-14 5.007026e-21 9.604924e-16 1.846807e-10 1.11688e-11 8.580568e-05 1.6899e-11 4.563676e-05 0.0008082298 0.006006504	**CRNDE** **XIST** **KIFC1**	3.762562 3.637404 2.133776	1.547281e-08 0.01229272 7.987507e-06

**Table 4:** The top 10 up- and down-regulated DEmiRNAs between GBM and normal samples.

Down-regulated			Up-regulated		
DEmiRNA	Log FC	Adjusted *P* value	DEmiRNA	Log FC	Adjusted P value
**hsa-miR-10b-5p** **hsa-miR-1290** **hsa-miR-371b-5p** **hsa-miR-199a-5p** **hsa-miR-21-3p** **hsa-miR-199a-3p** **hsa-miR-199b-3p** **hsa-miR-21-5p** **hsa-miR-431-5p** **hsa-miR-424-3p**	-4.7771098 -4.4993318 -4.4205838 -4.4046581 -4.274357 -4.1722017 -4.1722017 -3.9891862 -3.7024153 -3.5577576	0.00071 0.03931 0.005439 0.006768 0.000912 0.004278 0.004278 0.001823 0.001823 0.004493	**hsa-miR-219a-2-3p** **hsa-miR-338-5p** **hsa-miR-139-3p** **hsa-miR-383-5p** **hsa-miR-330-3p** **hsa-miR-584-5p** **hsa-miR-129-5p** **hsa-miR-330-5p** **hsa-miR-138-2-3p** **hsa-miR-1250-5p**	8.6479768 7.9211592 6.5955712 6.1274595 6.0482004 5.8478087 5.4260841 5.0897962 4.8481859 4.6317277	0.011486 0.000161 0.000161 0.001604 0.004003 0.007511 0.004825 0.000547 0.000161 0.003127

**Figure 3:**
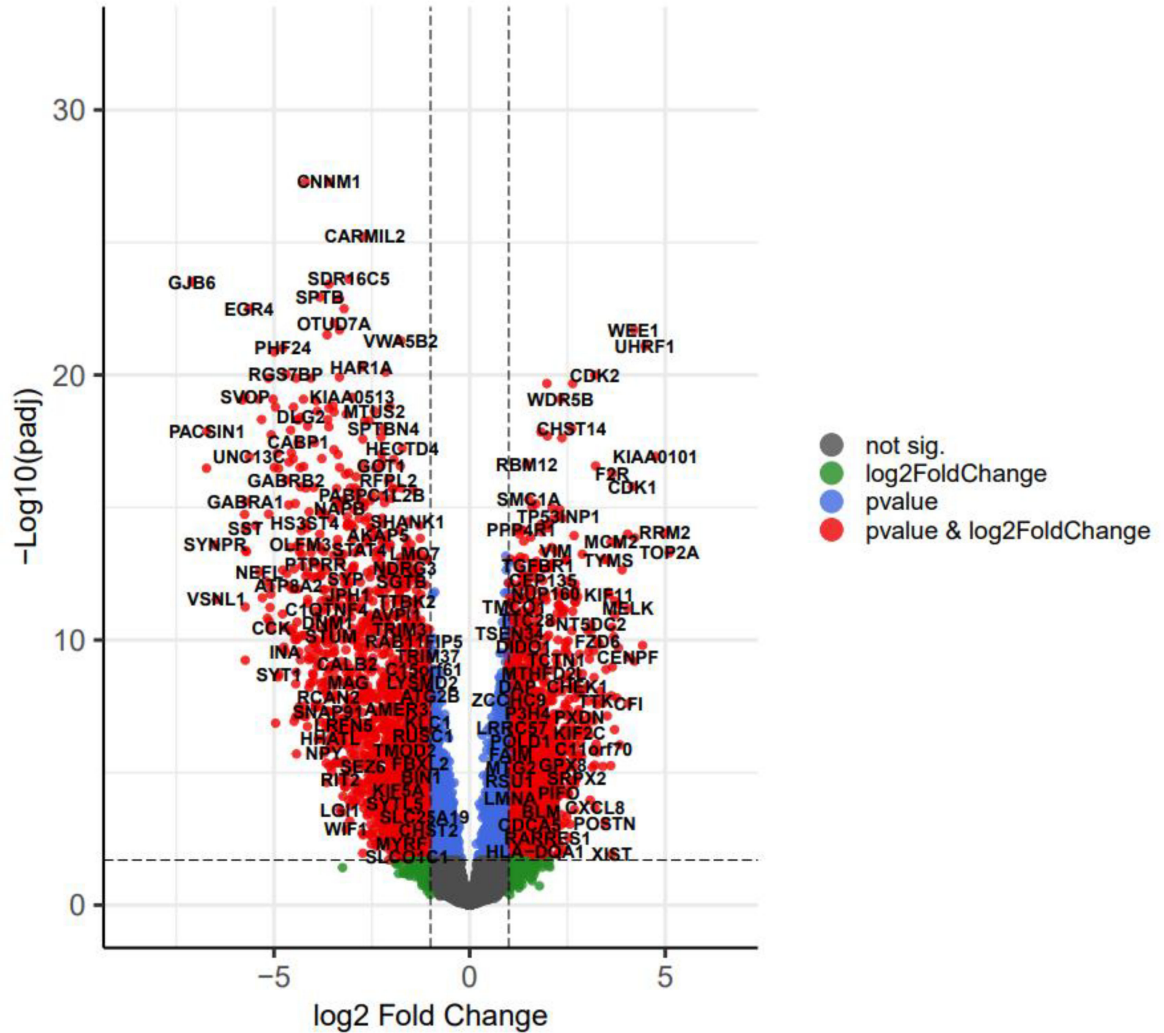
The volcano plot of differentially expressed genes (DEGs); horizontal axis, log_2_(FC); vertical axis, -log_10_(adjusted P value).

**Figure 4:**
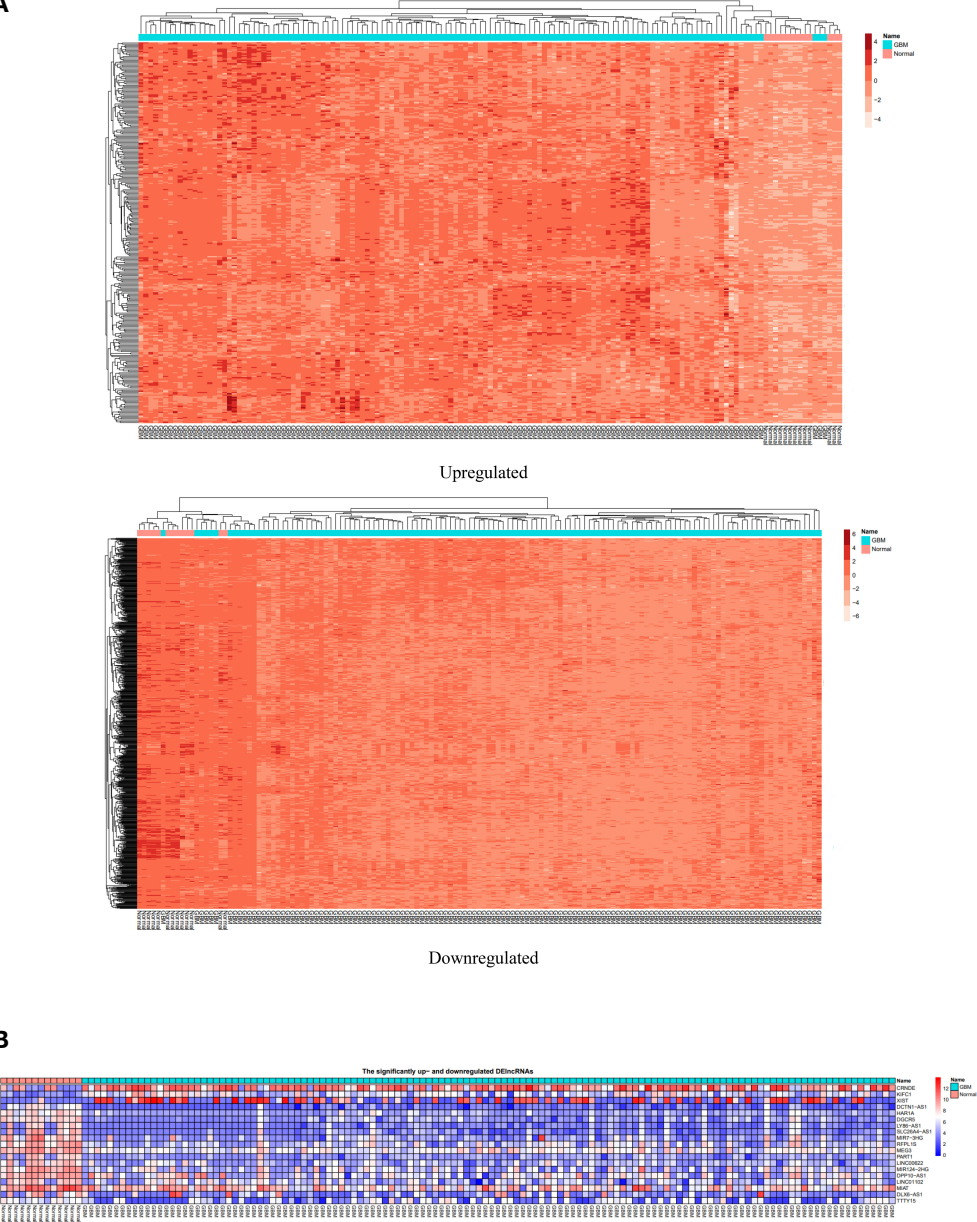
**(A)** The two-way clustering of DEmRNAs between GBM samples and normal samples; horizontal axis, the samples; vertical axis, DEmRNAs. **(B)** Two heatmaps depicting expression of DElncRNAs and DEmiRNAs.

We used the volcano plot with the Enhanced Volcano package (23) in R to compare the variation in miRNA, lncRNA, and mRNA expression between GBM and normal samples (**Figure 3**). In addition, the two-way clustering demonstrated that 20 clearly distinct DEmRNA expression patterns between GBM and normal samples were identifiable (**Figure 4A**). The expression of DElncRNAs and DEmiRNAs is also shown in two heatmaps (**Figure 4B**).

### GO enrichment analysis of DEGs

**Figure 5:**
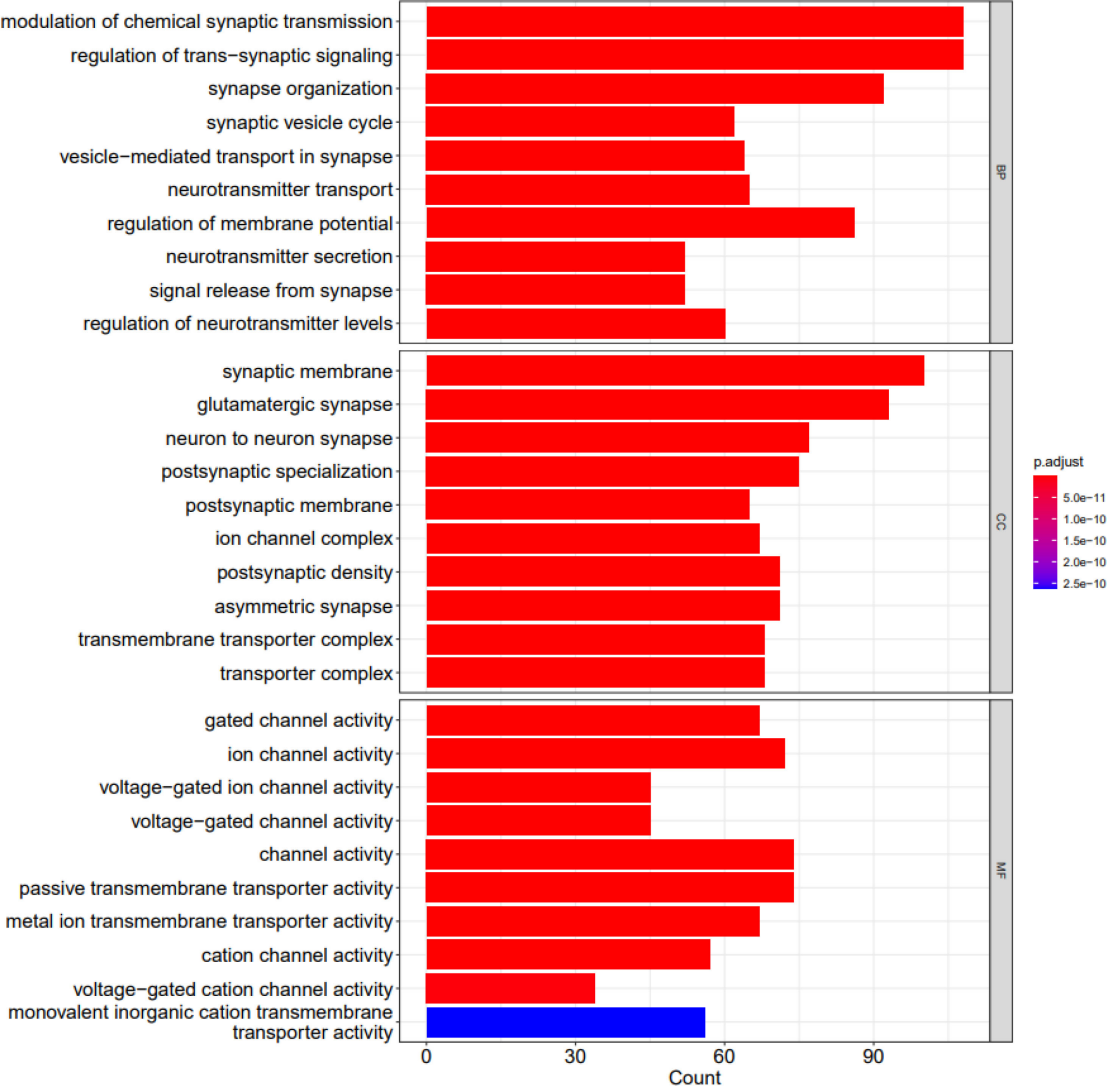
The barplots of top 10 enriched functions; BP (biological process), CC (cellular component) and MF (molecular function). X axis shows the count of geneset; Y axis shows the geneset function; Bar color represents the adjusted P.value, ranging from red (most significant) to blue (least significant).

DEGs were enriched in 816 GO terms. We used Clusterprofiler package to perform analysis. In GO functional enrichment analysis, 816 GO entries satisfy adjusted P value less than 0.05, most of which are biological processes (BP), followed by cellular components (CC) and molecular functions (MF). The first 30 entries are synaptic membrane (CC), modulation of chemical synaptic transmission (BP), regulation of trans-synaptic signaling (BP), glutamatergic synapse (CC), synapse organization (BP), synaptic vesicle cycle (BP), vesicle-mediated transport in synapse (BP), neurotransmitter transport (BP), regulation of membrane potential (BP), neuron to neuron synapse (CC), postsynaptic specialization (CC), postsynaptic membrane (CC), neurotransmitter secretion (BP), signal release from synapse (BP), ion channel complex (CC), regulation of neurotransmitter levels (BP), postsynaptic density (CC), asymmetric synapse (CC), transmembrane transporter complex (CC), transporter complex (CC), cation channel complex (CC), regulation of synaptic plasticity (BP), presynaptic membrane (CC), synaptic vesicle membrane (CC), exocytic vesicle membrane (CC), synaptic vesicle (CC), regulation of ion transmembrane transport (BP), exocytic vesicle (CC), synaptic vesicle exocytosis (BP) and gated channel activity (MF). **Figure 5** shows the barplots of top 10 enriched functions.

**Figure 6:**
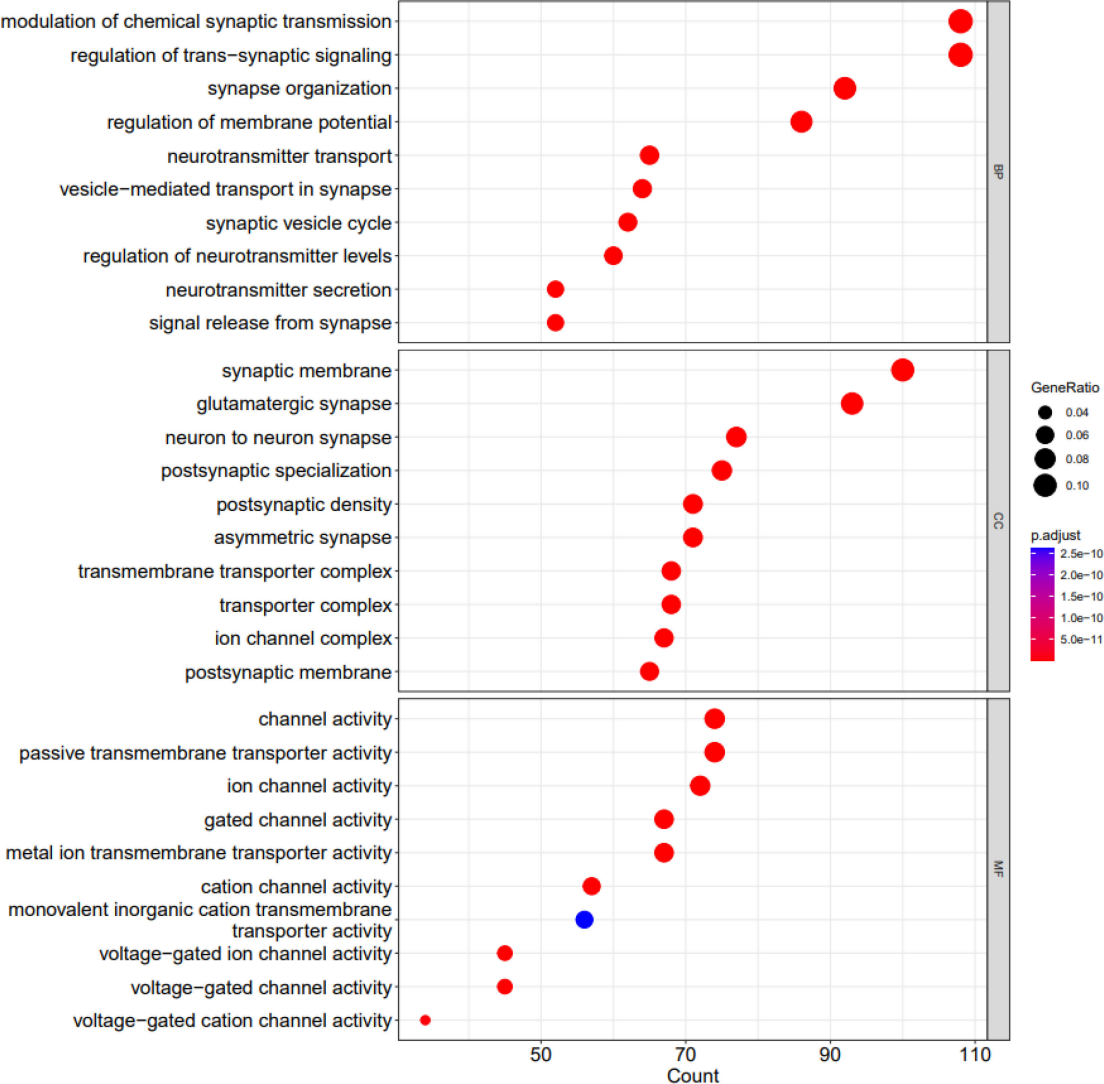
The dotplots of top 10 enriched functions. X axis shows the count of geneset; Y axis shows the geneset function; Dot color represents the adjusted P value ranging from dark blue (most significant) to red (least significant). Dot size represents the GeneRatio and the larger the size of the dot, the higher the value of the gene ratio.

**Figure 7:**
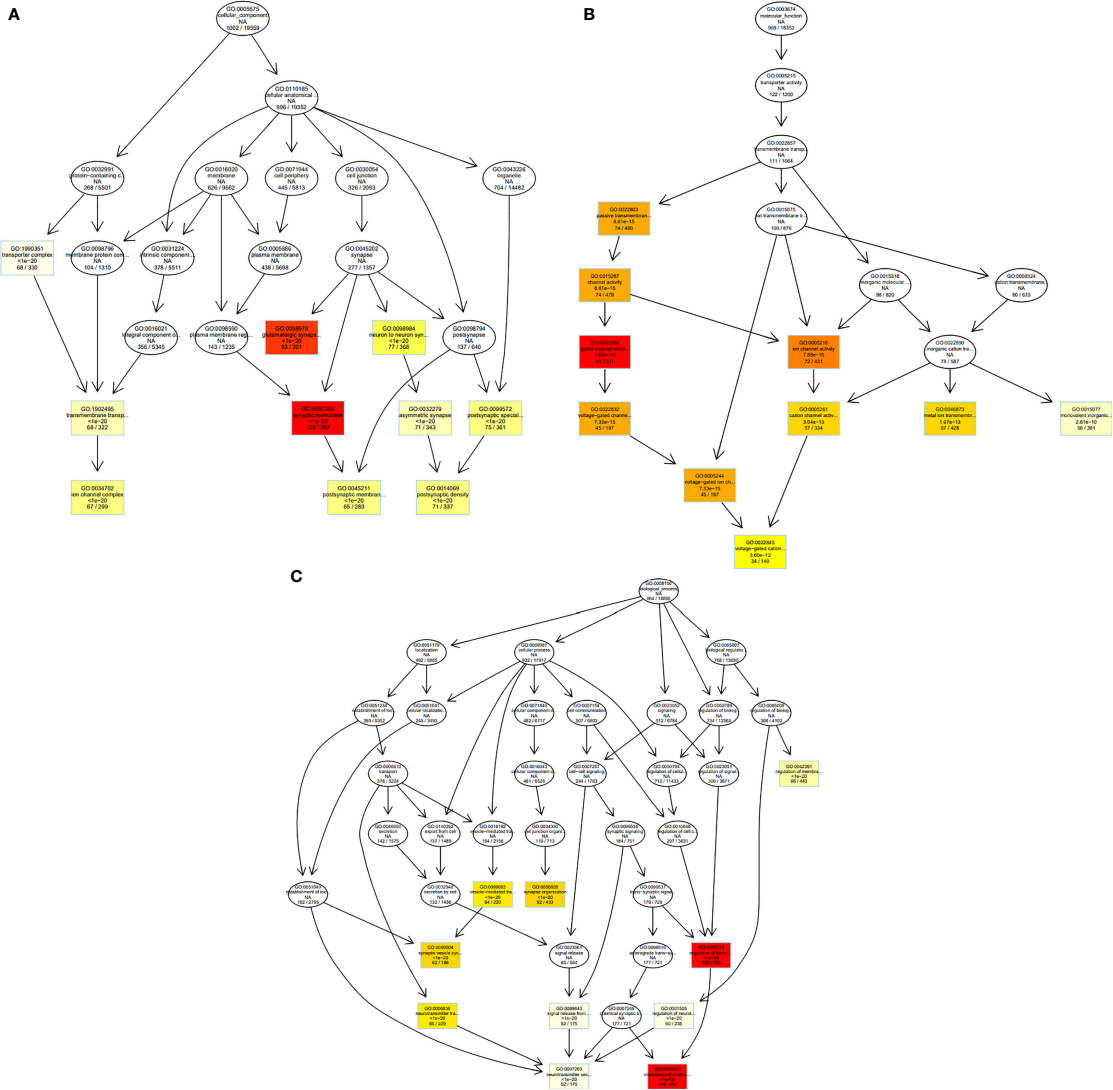
GO graph visualization of top GO terms enriched. **(A)** The top 10 GO terms in the category “Cellular Component” have generated a GO sub-graph. **(B)** The top 10 GO terms in the category “Molecular Function” have generated a GO sub-graph. **(C)** The top 10 GO terms in the category “Biological process” have generated a GO sub-graph. Boxes indicate the most significant terms. From dark red (most significant) to light yellow (least significant), the color of the box indicates the relative significance.

**Figures 6** and **7** depict visualization of the dotplots of top 10 enriched functions and enriched GO induced graph, respectively.

**Figure 8:**
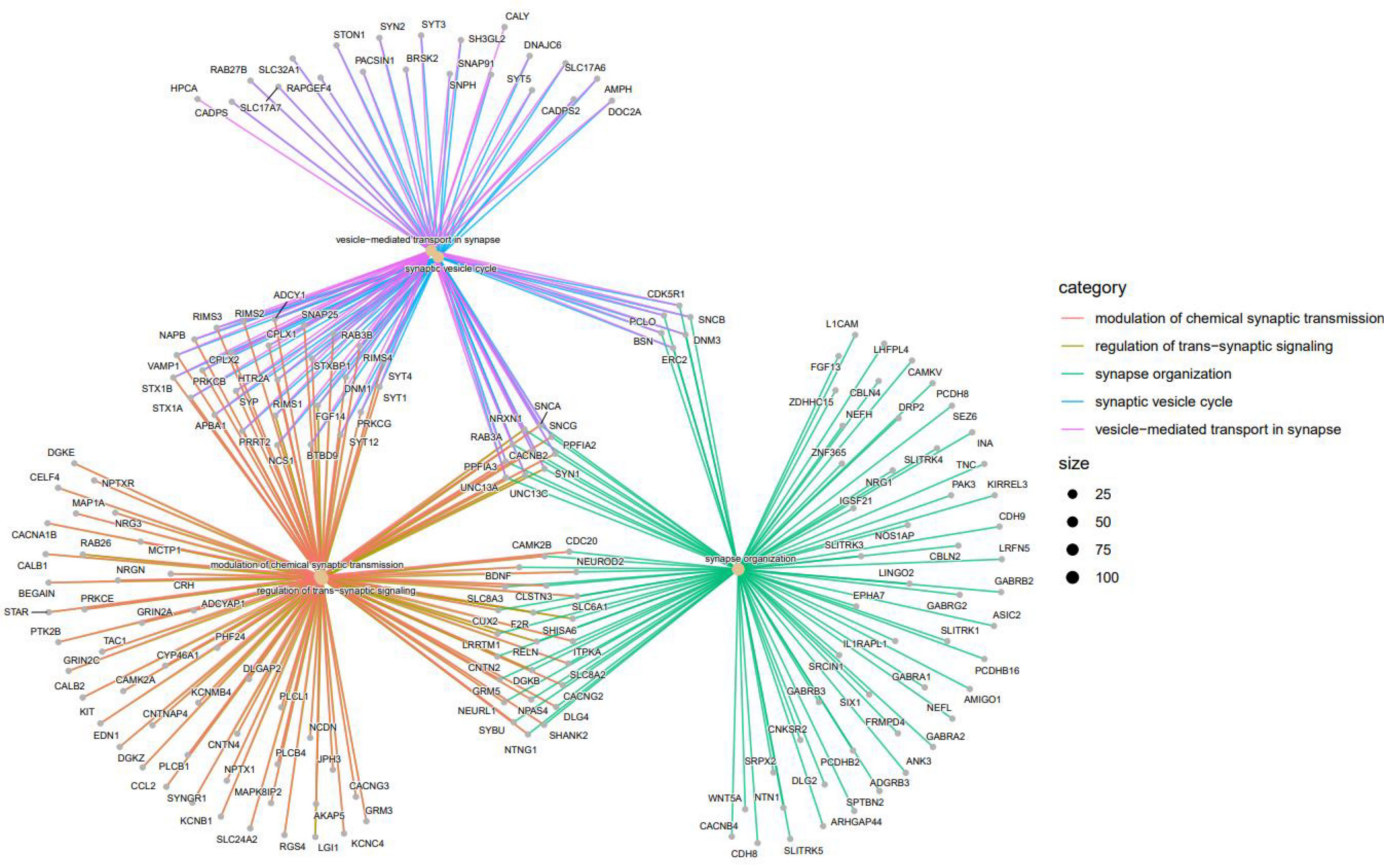
Top 5 GO terms as a network plot. These GO terms are connected to genes in this graph. The connection of genes to the corresponding GO is marked with a special color; There are more genes for a specific GO term if the dot relating to it is bigger.

**Figure 8** indicates the gene-concept network of top 5 GO terms (Modulation of chemical synaptic transmission, regulation of trans-synaptic signaling, synapse organization, synaptic vesicle cycle and vesicle-mediated transport in synapse).

**Figure 9:**
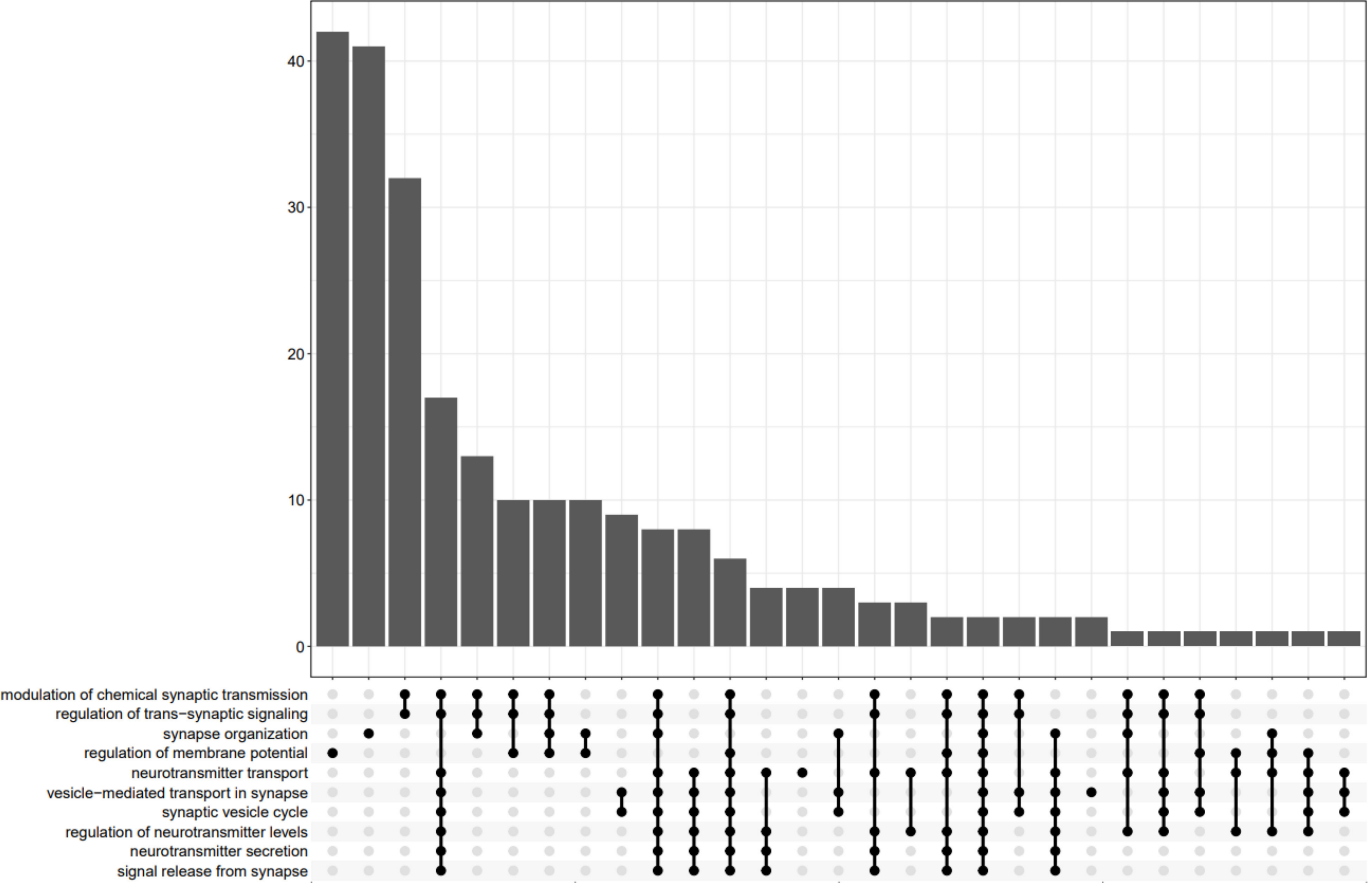
UpsetPlot of 10 GO terms.

In **Figure 9**, the UpSet plot visualizes the intersection between top 10 GO terms. It highlights the gene overlap between several gene sets.

### Pathway analysis

**Figure 10:**
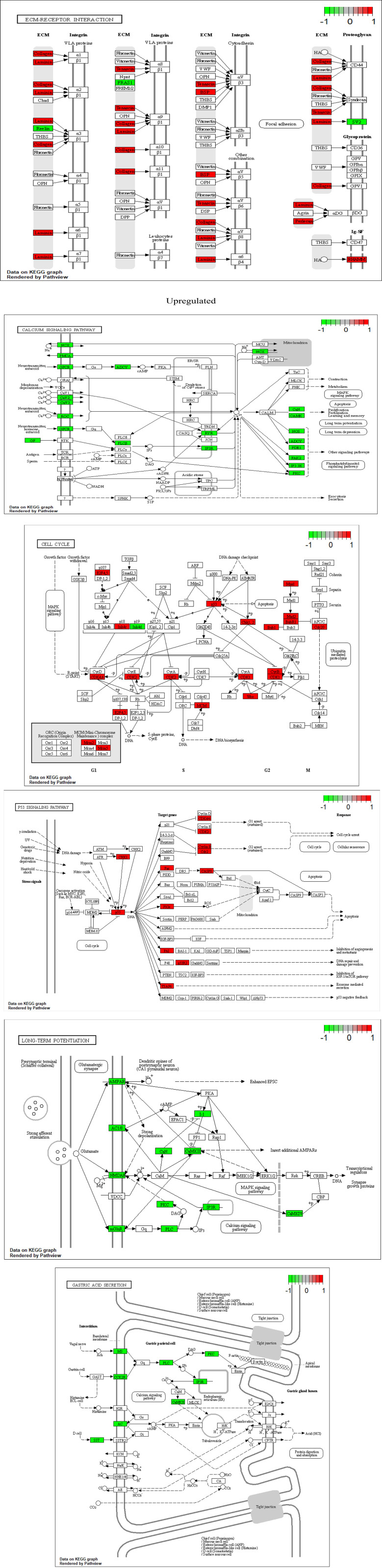
Visualization of the first two upregulated and downregulated pathways. Green boxes are downregulated genes and red boxes are upregulated genes.

Using Pathview (24) and gage (25) packages in R, KEGG pathways analysis of 177 downregulated and 177 upregulated DEGs were performed to identify the potential functional genes (**Table 5**; **Figure 10**).

**Table 5:** Down-regulated and Up-regulated Pathways.

Down-regulated		Up-regulated	
Pathway	P value	Pathway	P value
**Calcium signaling pathway** **Long-term potentiation** **Gastric acid secretion** **Pancreatic secretion**	0.002285132 0.018132471 0.025751413 0.034688834	**Cell cycle** **p53 signaling pathway** **ECM-receptor interaction** **Focal adhesion**	2.009266e-06 2.197253e-05 4.957866e-03 3.223350e-02

### PPI network construction and selection of hub genes

**Figure 11:**
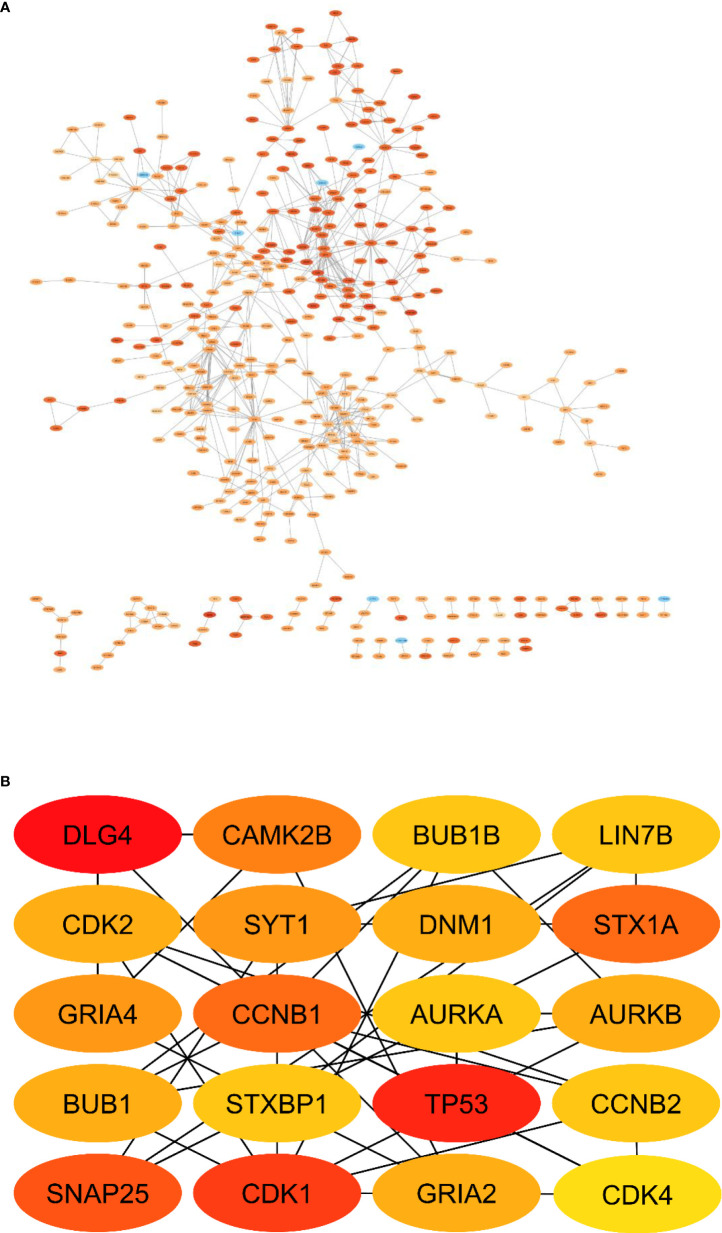
PPI network of DEmRNAs. **(A)** Total PPI network, **(B)** Subnetwork of hub genes.

A PPI network of DEGs (**Figure 11**) with 411 nodes and 727 edges that was generated from STRING was put into the Cytohubba plugin of Cytoscape 3.9 in order to identify the hub genes. The 20 hub genes with the highest dgree of connectivity were DLG4, CAMK2B, BUB1B, LIN7B, CDK2, SYT1, DNM1, STX1A, GRIA4, CCNB1, AURKA, AURKB, BUB1, STXBP1, TP53, CCNB2, SNAP25, CDK1, GRIA2 and CDK4. **Table 6** is a list of this hub’s information. The greatest degree to lowest degree is used to order these hubs.

**Table 6:** The information of hub genes in PPI network.

Hub Gene	Adjusted P.value	Log2FC	Clustering Coefficient	Degree	Closseness Centrality	Betweenness Centrality
**DLG4** **TP53** **CDK1** **SNAP25** **CCNB1** **STX1A** **CAMK2B** **GRIA4** **SYT1** **AURKB** **BUB1** **CDK2** **DNM1** **GRIA2** **AURKA** **BUB1B** **CCNB2** **LIN7B** **STXBP1** **CDK4**	2.36E-15 1.87E-12 1.64E-16 1.34E-07 2.23E-06 1.45E-15 8.26E-12 0.001527989 1.99E-09 1.88E-05 6.85E-07 1.01E-20 2.27E-11 0.002650131 2.22E-07 3.32E-11 3.40E-12 2.52E-11 6.03E-14 1.08E-11	-2.598617544 2.464542358 4.164108388 -3.669305973 2.248216201 -4.103297234 -3.073502871 -2.209019827 -4.875013677 2.02463802 2.835182173 3.199041769 -3.62934314 -2.194510124 2.456079967 3.639344188 3.703784989 -2.039148743 -2.263599416 2.368774869	0.071428571 0.043333333 0.204761905 0.205263158 0.267973856 0.196078431 0.352380952 0.384615385 0.318681319 0.179487179 0.358974359 0.358974359 0.128205128 0.294871795 0.136363636 0.363636364 0.424242424 0.484848485 0.484848485 0.436363636	28 25 21 20 18 18 15 14 14 13 13 13 13 13 12 12 12 12 12 11	0.224764468 0.242556282 0.236376504 0.207840697 0.210327456 0.195321637 0.212603437 0.207970112 0.19716647 0.214515093 0.203039514 0.21618123 0.166003976 0.206427689 0.234057463 0.20291616 0.203410475 0.187640449 0.192618224 0.214652956	0.198126639 0.292493307 0.17080442 0.082044934 0.031022432 0.050648257 0.034827756 0.02887363 0.049932638 0.060609355 0.020336039 0.028807346 0.082152881 0.021606876 0.122769569 0.017593973 0.00663464 0.010884111 0.015683204 0.010245679

### CeRNA network construction in GBM

**Figure 12:**
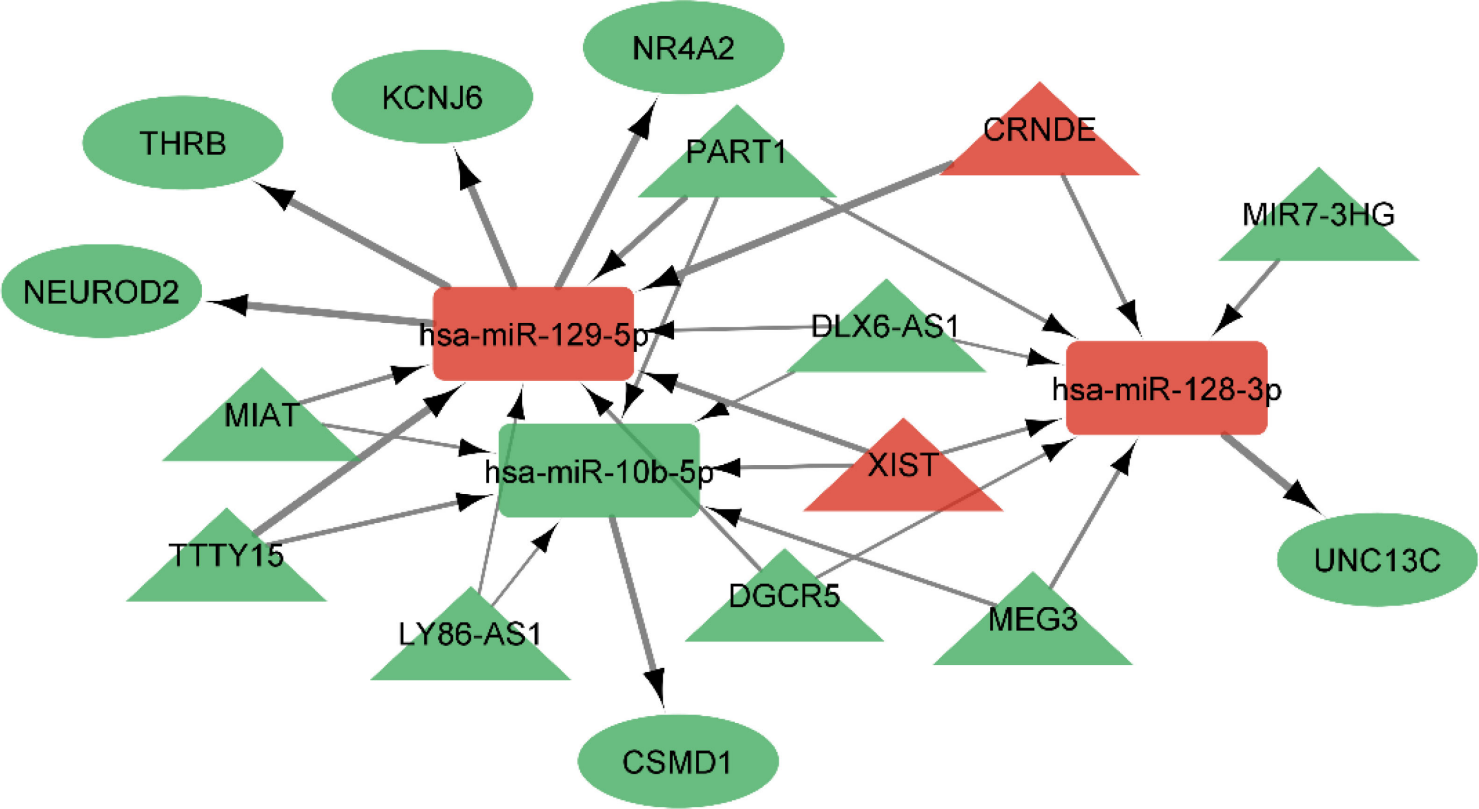
ceRNA network in GBM. Red nodes signify a strong expression level, while green nodes signify a low level of expression. Ellipses represent protein-coding genes; rectangles represent miRNAs; Triangles represent lncRNAs; gray edges indicate lncRNA-miRNA-mRNA interactions. Greater edge thickness indicates greater betweenness.

**Figure 13:**
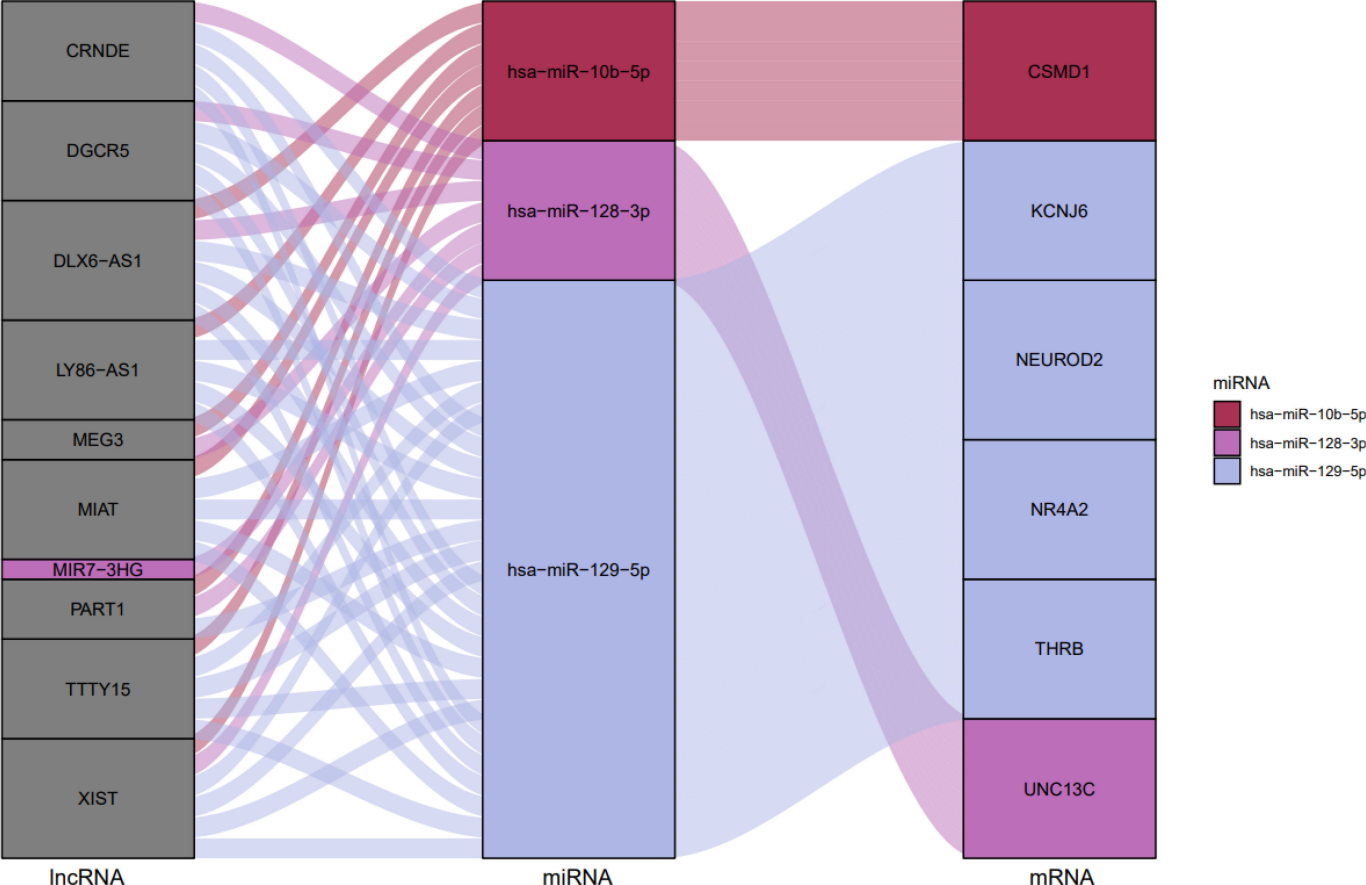
The ceRNA network in GBM is shown by a Sankey diagram. Each rectangle represents a gene, and depending on the size of the rectangle, the degree of relationship between each gene is shown.

**Figure 14:**
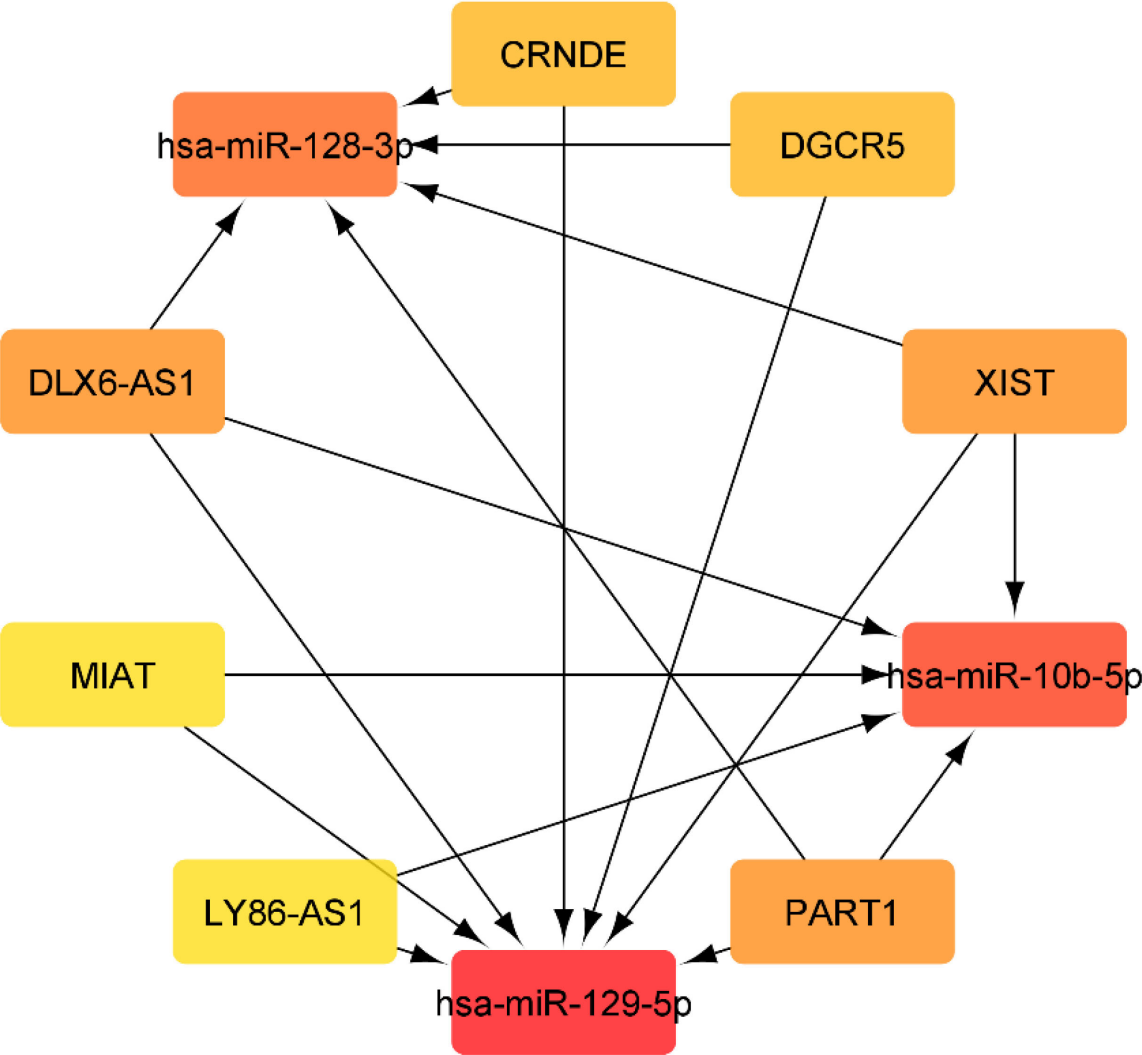
Top 10 genes with highest closeness centrality in ceRNA network.

Using miRcode, the relationship between lncRNAs and miRNAs was assessed. This step demonstrated that 20 of the 39 GBM-specific DEmiRNAs may be targeted by 10 of the 19 lncRNAs (**Table 7**). Then, in order to investigate the connection between miRNAs and mRNAs, we used miRWalk with miRTarBase, TargetScan and miRDB filters to predict targeted mRNAs by these 20 miRNA. The findings suggested that 3 miRNAs may target 6 of the 1080 mRNAs (**Table 8**). If miRNA-targeted mRNAs were not found in DEmRNAs, they were eliminated. Cytoscape 3.9 was used to build the lncRNA-miRNA-mRNA ceRNA network using the data from **Tables 7** and **8**. The ceRNA network contained a total of 3 miRNAs, 10 lncRNAs, and 6 mRNAs (**Figure 12**). We displayed this ceRNA network using a Sankey diagram generated by the ggalluvial R package (Version: 0.12.3) (26) in order to better understand the impact of lncRNAs on mRNAs in GBM that is mediated by their interaction with miRNAs (**Figure 13**). Finally, using the cytohubba app, we calculated nodes closeness and exhibited the top 10 nodes in the network with the highest closeness centrality (**Figure 14**). We identified DGCR5, MIAT, hsa-miR-129-5p, XIST, hsa-miR-128-3p, PART1, hsa-miR-10b-5p, LY86-AS1, CRNDE, and DLX6-AS1 as 10 hub genes in the ceRNA network.

**Table 7:** The MiRcode database revealed interactions between 10 DElncRNAs and 20 DEmiRNAs.

lncRNA	miRNA
CRNDE, XIST, DGCR5, MEG3, MIAT, DLX6-AS1	hsa-miR-338-5p
CRNDE, XIST, MEG3, MIAT	hsa-miR-1244
CRNDE, XIST, DGCR5, MIR7-3HG, MEG3, PART1, DLX6-AS1	hsa-miR-128-3p
CRNDE, XIST, DGCR5, LY86-AS1, PART1, MIAT, DLX6-AS1, TTTY15	hsa-miR-129-5p
CRNDE, XIST, DGCR5, MEG3, MIAT, DLX6-AS1	hsa-miR-338-3p
CRNDE, XIST, DGCR5, LY86-AS1, MEG3, MIAT, DLX6-AS1	hsa-miR-23b-5p
CRNDE, DGCR5, MEG3, PART1, MIAT, DLX6-AS1	hsa-miR-199a-5p
XIST, DGCR5, LY86-AS1, MIR7-3HG, MEG3, PART1	hsa-miR-138-2-3p
XIST, DGCR5, LY86-AS1, MIAT, DLX6-AS1, TTTY15	hsa-miR-29c-5p
XIST, DGCR5, LY86-AS1, MEG3, DLX6-AS1,	hsa-miR-383-5p
XIST, LY86-AS1, MIAT, DLX6-AS1, TTTY15	hsa-miR-139-5p
XIST, DGCR5, LY86-AS1, MEG3, PART1, MIAT	hsa-miR-34c-3p
XIST, DGCR5, LY86-AS1, MIAT, DLX6-AS1, TTTY15	hsa-miR-29b-2-5p
XIST, LY86-AS1, MEG3, PART1, MIAT, DLX6-AS1, TTTY15	hsa-miR-10b-5p
XIST, DGCR5, MEG3, PART1, TTTY15	hsa-miR-21-3p
XIST, DGCR5, MEG3, PART1, TTTY15	hsa-miR-21-5p
XIST, DGCR5, LY86-AS1, MEG3, PART1, MIAT, DLX6-AS1	hsa-miR-424-3p
LY86-AS1, MIAT	hsa-miR-139-3p
LY86-AS1, MEG3, MIAT	hsa-miR-184
LY86-AS1	hsa-miR-129-2-3p

**Table 8:** miRWalk (miRTarBase, TargetScan and miRDB filters) database revealed interactions between 3 DEmiRNAs and 6 DEmRNAs.

miRNA	mRNA
hsa-miR-128-3p	UNC13C
hsa-miR-129-5p	NEUROD2, NR4A2, THRB, KCNJ6
hsa-miR-10b-5p	CSMD1

### Validation of hub genes *via* expression value

**Figure 15:**
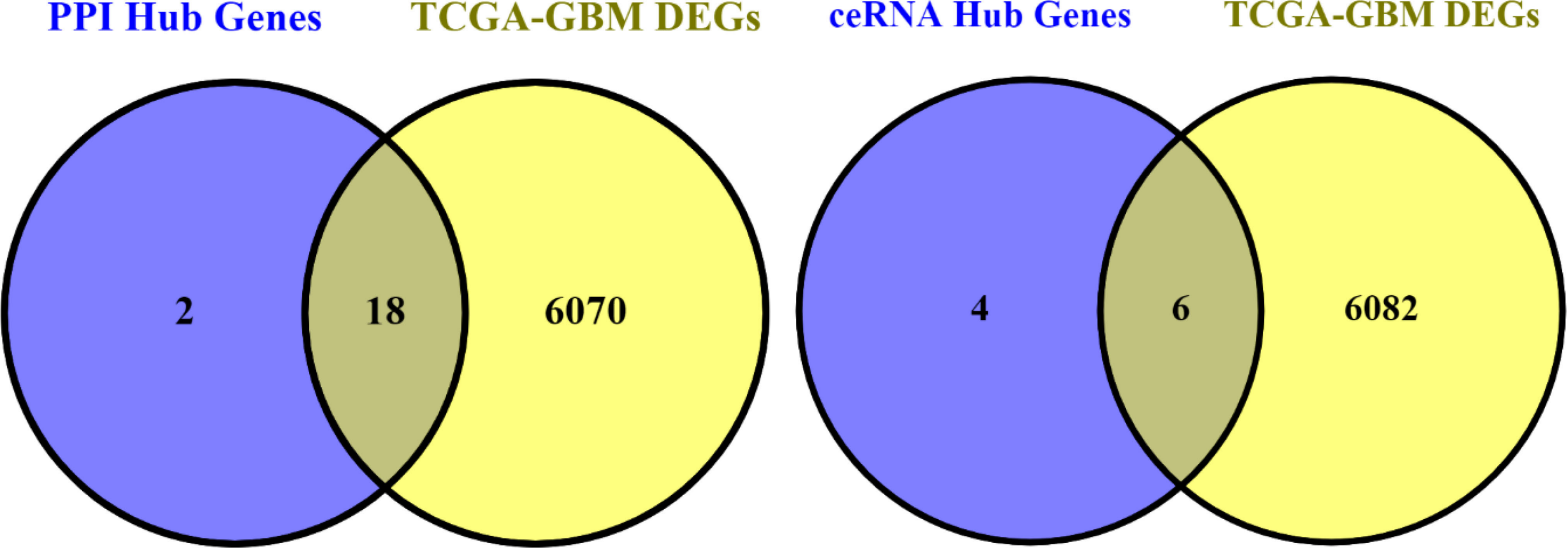
Venn diagram. The number of hub genes in PPI and ceRNA networks and TCGA-GBM DEGs.

**Figure 16:**
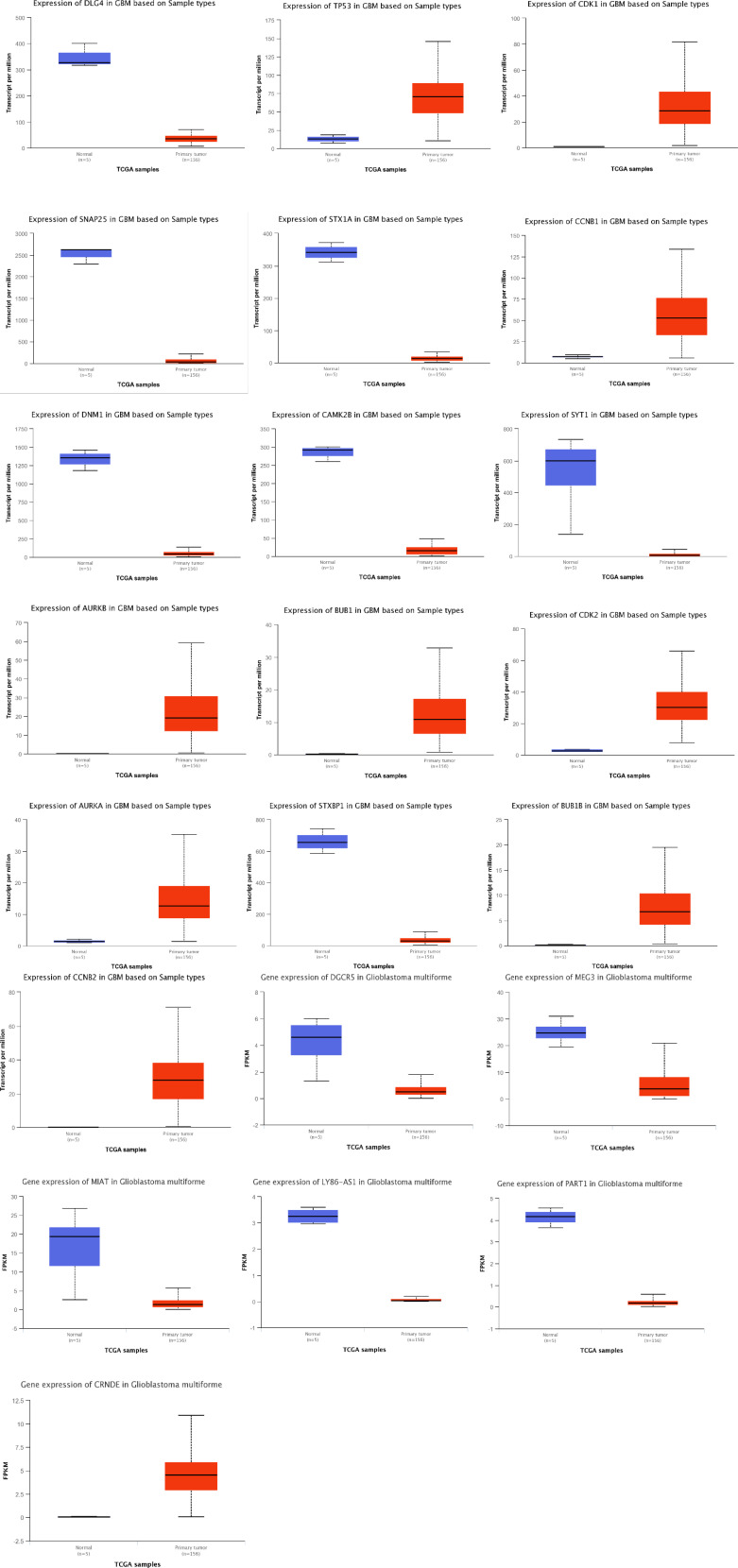
Box plots of gene expression of hub genes in GBM and normal samples based on TCGA. Red and blue boxes show gene expression of hub genes in GBM and normal samples, respectively.

We first obtained the TCGA-GBM RNA-seq data using the TCGAbiolinks package (27), and then we used the R packages limma and edgeR to analyze it. Then, by using Venny 2.0.2 (28), we were able to obtain the genes that were present in both the PPI and ceRNA networks’ Hub genes and TCGA-GBM DEGs (adjusted p value < 0.05 and |log2 fold Change (FC)| > 2) (**Figure 15**). As a result, 18 of the 20 PPI network genes and 6 of the 10 ceRNA network genes were also included in the TCGA-GBM DEGs. Utilizing the ualcan, the expression value of these hub genes was evaluated. Therefore, all hub genes in PPI network and CRNDE, DGCR5, LY86-AS1, MEG3, MIAT, PART1 in ceRNA network revealed excellent statistical significance (**Figure 16**; **Table 9**).

**Table 9:** Statistical significance of hub genes based on sample types in GBM.

Hub genes	Statistical significance of expression value*
DLG4	4.066400E-02
TP53	1.62625468647093E-12
CDK1	1.62436730732907E-12
SNAP25	3.176000E-02
STX1A	4.005600E-02
CCNB1	<1E-12
CAMK2B	3.325700E-02
SYT1	3.254000E-02
DNM1	3.230500E-02
AURKB	1.62447832963153E-12
BUB1	<1E-12
CDK2	1.62447832963153E-12
AURKA	<1E-12
STXBP1	3.203700E-02
BUB1B	1.62447832963153E-12
CCNB2	1.62447832963153E-12
APBA1	2.727800E-02
DGCR5	2.698251E-02
MEG3	4.831681E-02
MIAT	3.234847E-02
LY86-AS1	1.431129E-02
PART1	1.269176E-02
CRNDE	3.024283719995E-31

*Low number (<10) of normal samples considered.

### Expression of the hub genes in various GBM cell lines

Using the cancer cell line encyclopedia (CCLE), we gathered cell line expression data (DepMap Public 22Q2) (29) and chose four GBM cell lines and hub genes. We selected four primary cell lines for GBM, namely A172, U251, U87, and T98G. NHA cell line was utilized as a normal brain cell line. Finally, we analyzed this data using the limma package in the R programming language, and we discovered how the hub genes are expressed in diverse GBM cell lines (**Table 10**). As a threshold, we utilized Log2FC > |0.5| and an adjusted P.value of 0.05.

**Table 10:** Expression pattern of the hub genes in A172, U251, U87, and T98G cell lines.

Hub gene	A172	U87	U251	T98G
DLG4	Not significant	Downregulated	Downregulated	Downregulated
TP53	Not significant	Downregulated	Upregulated	Upregulated
CDK1	Upregulated	Upregulated	Upregulated	Upregulated
SNAP25	Not significant	Upregulated	Downregulated	Downregulated
STX1A	Not significant	Upregulated	Downregulated	Downregulated
CCNB1	Upregulated	Upregulated	Downregulated	Upregulated
CAMK2B	Upregulated	Downregulated	Downregulated	Downregulated
SYT1	Not significant	Downregulated	Downregulated	Downregulated
DNM1	Not significant	Upregulated	Upregulated	Downregulated
AURKB	Upregulated	Upregulated	Upregulated	Upregulated
BUB1	Upregulated	Upregulated	Upregulated	Upregulated
CDK2	Upregulated	Upregulated	Upregulated	Upregulated
AURKA	Not significant	Upregulated	Upregulated	Upregulated
STXBP1	Not significant	Upregulated	Downregulated	Downregulated
BUB1B	Upregulated	Upregulated	Downregulated	Upregulated
CCNB2	Upregulated	Upregulated	Upregulated	Upregulated
APBA1	Not significant	Downregulated	Upregulated	Downregulated

### Survival analysis

**Figure 17:**
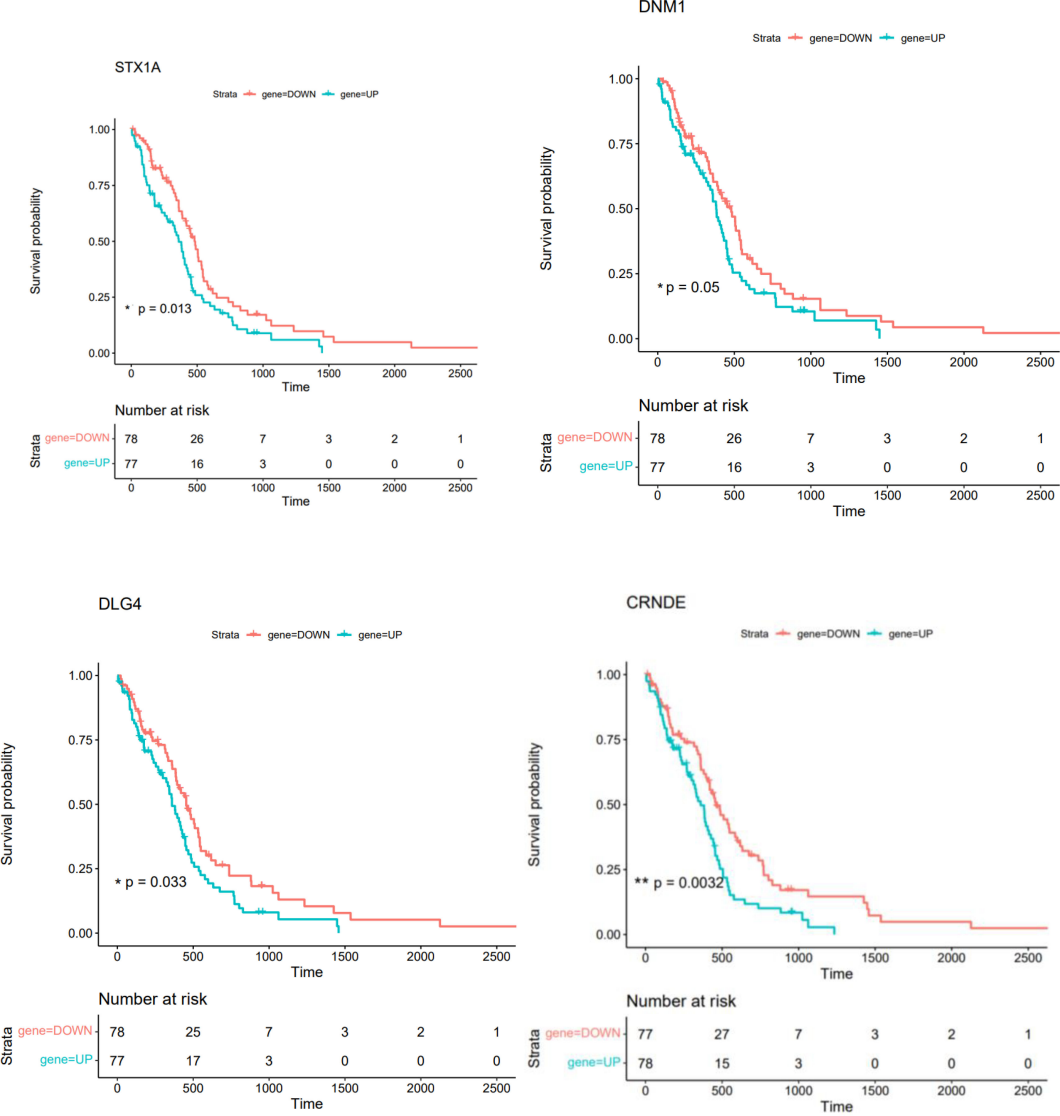
The overall survival of GBM patients is related to the Kaplan-Meier survival curves of STX1A, DNM1, DLG4, and CRNDE (* shows P<=0.05, ** shows P<0.01).

A Kaplan-Meier curve analysis was used to perform a survival analysis using the R survival package. We carried out a survival analysis using hub genes in PPI and ceRNA networks. The difference was statistically significant with a log-rank P value less than 0.05. Therefore, in patients with GBM, DLG4, DNM1, STX1, and CRNDE exhibited a significant correlation with a shorter overall survival time (**Figure 17**).

## Discussion

Using an *in-silico* approach, we aimed to identify ceRNA networks in GBM. The ceRNA network between three mentioned classes of RNAs is a recently revealed regulatory relationship. This network has an essential role in the modulation of biological features of cancer. Our strategy led to identification of 1080 DEmRNAs, including 777 downregulated DEmRNAs (such as GJB6 and SLC12A5) and 303 upregulated DEmRNAs (such as TOP2A and RRM2), 19 DElncRNAs, including 16 downregulated DElncRNAs (such as MIR7-3HG and MIR124-2HG) and 3 upregulated DElncRNAs (such as CRNDE and XIST) and 49 DEmiRNAs, including 10 downregulated DEmiRNAs (such as hsa-miR-10b-5p and hsa-miR-1290) and 39 upregulated DEmiRNAs (such as hsa-miR-219a-2-3p and hsa-miR-338-5p).

In line with our results, a previous study has shown that lncRNA XIST has as oncogenic function in human glioma through influencing expression of miR-137 (30). Moreover, ceRNA network analyses have shown that CRNDE enhances glioblastoma progression *via* sponging miR-9-5p (31).

Modulation of chemical synaptic transmission, regulation of trans-synaptic signaling, synapse organization, synaptic vesicle cycle and vesicle-mediated transport in synapse were identified as the top five GO terms. Therefore, the most important pathways are related with synaptic function.

DGCR5, MIAT, hsa-miR-129-5p, XIST, hsa-miR-128-3p, PART1, hsa-miR-10b-5p, LY86-AS1, CRNDE, and DLX6-AS1 were identified as 10 hub genes in the ceRNA network. These different types of RNAs are possible therapeutic targets and markers for GBM. Further experiments revealed association between expression of DLG4, DNM1, STX1, and CRNDE and overall survival time of GBM patients, indicating their importance as prognostic factors. DLG4 has been identified as a core biomarker Biomarkers related with clinical outcome in glioma patients through a bioinformatics approach (32). Bioinformatics analyses have also identified DNM1 as a marker of invasion in this type of cancer (33). Besides, functional studies have shown that interference with the Stx1 function can impair progression of GBM *in vivo*.

Additionally, a prior study has shown that blocking the SNARE protein Stx1 *via* three distinct methods, including STX1A knockdown, consistently results in a marked slowing of the growth of glioblastoma tumors in an orthotopic mouse model (34).

According to reports, DNM1 promotes the growth of tumors in a number of malignancies, including gastric adenocarcinoma (35). We also found DNM1 to be a significant biomarker in GBM.

DLG4, which was also recognized as a key gene hub in this illness was another gene that we discovered to be a biomarker in GBM (36).

Taken together, ceRNA network analyses in GBM have provided new insights into molecular mechanisms in this type of cancer, representing novel markers and therapeutic targets in GBM. Future assessment of their expression in clinical samples and functional studies in animal models would lead to identification of detailed data in this regard.

## Data availability statement

Publicly available datasets were analyzed in this study. This data can be found here: https://www.ncbi.nlm.nih.gov/geo/(GSE50161, GSE36245, GSE83300 and GSE65626).

## Author contributions

SG-F wrote the draft and revised it. GS and MT designed and supervised the study. BH, AS, and MA-B performed the bioinformatic analysis and data collection. All authors contributed to the article and approved the submitted version.

## Funding

The authors would like to thank the clinical Research Development Unit (CRDU) of Loghman Hakim Hospital, Shahid Beheshti University of Medical Sciences, Tehran, Iran for their support, cooperation and assistance throughout the period of study (Grant Number 33186).

## Conflict of interest

The authors declare that the research was conducted in the absence of any commercial or financial relationships that could be construed as a potential conflict of interest.

## Publisher’s note

All claims expressed in this article are solely those of the authors and do not necessarily represent those of their affiliated organizations, or those of the publisher, the editors and the reviewers. Any product that may be evaluated in this article, or claim that may be made by its manufacturer, is not guaranteed or endorsed by the publisher.

## Abbreviations

GBM, Glioblastoma Multiforme; lncRNA, Long non-coding RNA; miRNA, MicroRNA; mRNA, Messenger RNA; ceRNA, Competitive Endogenous RNA; DEG, Differentially Expressed Genes; GEO, Gene Expression Omnibus; SVA, Surrogate Variable Analysis; PCA, Principal Component Analysis; Limma, Linear Models for Microarray Data; FDR, False Discovery Rate; Log2FC, Log2 Fold Change; HGNC, HUGO gene nomenclature; GO, Gene Ontology; KEGG, Kyoto Encyclopedia of Genes and Genomes; PPI, Protein-Protein Interaction; TCGA, The Cancer Genome Atlas; BP, Biological Process; MF, Molecular Function; CC, Cellular Component; CCLE, Cancer Cell Line Encyclopedia.
